# Modeling the Influence of Nonclinic Visits on the Transmission of Respiratory Diseases

**DOI:** 10.1155/2020/8049631

**Published:** 2020-05-31

**Authors:** Yunting Bao, Yanlong Xu, Longxing Qi, Sulan Zhai

**Affiliations:** ^1^School of Mathematical Sciences, Anhui University, Hefei 230601, China; ^2^Anhui Provincial Center for Disease Control and Prevention, Hefei 230601, China

## Abstract

According to the information reflected by Anhui Center for Disease Control (Anhui CDC) in Hefei, Anhui province of China, some patients infected with respiratory diseases did not seek medical treatment (nonclinic visits) due to their strong resistance, and the influence of them on the spread of respiratory diseases has not been known. A SIS model with considering the nonclinic visits was established; a qualitative theory of the model was analyzed to obtain the basic reproduction number *R*_0_, disease-free equilibrium, endemic equilibrium, and stability of two equilibriums. Then, the model is combined with the daily number of respiratory diseases for parameter estimation and numerical simulation. Numerical simulation results showed that respiratory diseases were easy to break out in the autumn and winter and were relatively stable in the spring and summer. Through parameter estimation, the unknown parameter value was achieved and the result was obtained that the initial number of nonclinic visits is 10-11 times that of clinic visits. Finally, the result of sensitivity analysis displayed that the proportion of the number of nonclinic visits to the total number of patients has a significant influence on the final number of patients. If persons improve their resistance so that the number of nonclinic visits increases, the total number of patients will be reduced or even reduced to zero. Besides, reducing contact infection rate of disease and increasing the cure rate can also reduce the final total number of patients.

## 1. Introduction

**Figure 1:**
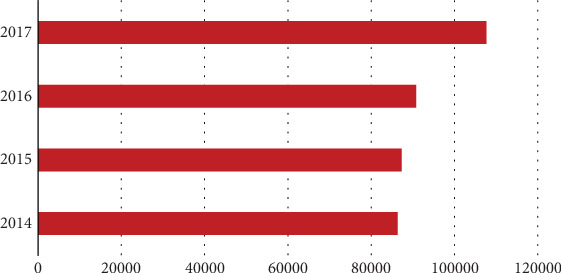
Annual cases of respiratory diseases (the data from Anhui CDC).

In recent years, the respiratory diseases in China have become increasingly serious. This article takes Hefei city in Anhui Province as an example to research.  Figure 1 shows annual cases of respiratory diseases in Baohe District and Yaohai District of Hefei city, Anhui Province of China, from 2014 to 2017, with data from Anhui Provincial Center for Disease Control and Prevention (Anhui CDC). As can be observed in the figure, the number of cases of respiratory diseases increased year by year from 2014 to 2017, and the growth rate in 2017 was relatively large, so we need to pay more attention to the development of respiratory diseases.

Respiratory diseases are mainly transmitted to healthy people by droplets [1]. If respiratory patients seek medical treatment in time when they get sick, they can be isolated as soon as possible to reduce the force of transmission. However, patients with various types of respiratory diseases can be cured by their own immunity, such as acute upper respiratory tract infection and bronchitis [2, 3]. These patients may not choose to seek medical treatment, but they are contagious and may infect healthy people into new patients [4]. Patients who do not seek medical treatment is defined as *nonclinic visits* and those who seek medical treatment as *clinic visits*. Nonclinic visits contain asymptomatic patients and symptomatic patients and can be recovered by their resistance. We can acquire specific data on the number of respiratory disease patients in Hefei from Anhui CDC. However, it is unclear if there are a large number of nonclinic visits. The increasing of nonclinic visits may cause a significant influence in the transmission of respiratory diseases. It is necessary to conduct further research on the impact of nonclinic visits on respiratory diseases.

Nowadays, the outbreak of infectious respiratory diseases attracts worldwide attention. A lot of studies have researched on infectious respiratory diseases with respect to SARS, tuberculosis, seasonal influenza, and other diseases. According to the spread of SARS in Beijing, Chen et al. [5] divided residents into four categories: susceptible, suspected infected, infected, and recovered. Then, an SEIR transmission model was established, the parameters and initial values of the model were estimated, and a simulation experiment of SARS in Beijing was carried out. Wang and Ruan [6] divided the population into 6 groups: susceptible, exposed, isolated, suspected infected, possibly infected, and transferred according to the SARS transmission situation in Beijing, and established the SARS transmission model. Through combining the model with a SARS data in Beijing, the result showed that important control measures should be increased before reaching the threshold of the number of possible infected cases. By considering the total number of individuals recovered from nature or due to vaccination, a tuberculosis transmission model with vaccination was proposed by Nainggolan et al. [7]. Dynamics analysis of the model showed that vaccination could reduce the number of people infected later. For seasonal influenza, Kharis and Arifudin [2] added the participants to the treatment group based on SIR, established the SITR model, and divided the way for group I to the group R into two types: therapeutic rehabilitation and self-healing rehabilitation. Moreover, it was proposed that seasonal influenza rarely cause death, and the minimum proportion of patients to be treated was obtained through the basic regeneration number. Hsu and Hsieh [8] proposed a dynamics model that includes asymptomatic infection and symptomatic infection. Symptomatic-infected people recovered by treatment or their resistance. The results mainly clarified that asymptomatic infections have a positive or negative impact on the disease, but the specific impact of the nonclinic visits on the disease was not investigated.

People infected by infectious respiratory diseases such as bronchitis and influenza will have mild symptoms and can recover without medical treatment [2], and the recovery rate of clinic visits is not the same as that of nonclinic visits. However, the available monitoring data are all clinic visits in the research process. Most people with mild symptoms do not go to the hospital for medical treatment. Therefore, the data recorded by the hospital are not the total number of people infected with respiratory disease, and the number of nonclinic visits is unknown.

Motivated by Kharis and Hsu, we considered the infectivity of the nonclinic visits based on the number of respiratory disease cases in Baohe District and Yaohai District of city. The impact of nonclinic visits on the spread of respiratory disease is our main study. In addition, the number of nonclinic visits at the initial stage of the disease outbreak and the impact of nonclinic visits on the final total number of patients through the available data were also estimated.

In the second section of this paper, the model is established, and *R*_0_, equilibrium points are calculated. The third section analyzes the local and global stability of disease-free and endemic equilibrium. In the forth section, we use the MCMC method for parameter estimation and numerical simulation. The sensitivity of each parameter to the number of patients visited is obtained through PRCC sensitivity analysis and then studies the influence of the most sensitive parameter on the final total number of patients. The fifth part discusses the results and puts forward suggestions.

## 2. Model

### 2.1. Establishment of a Model

**Figure 2:**
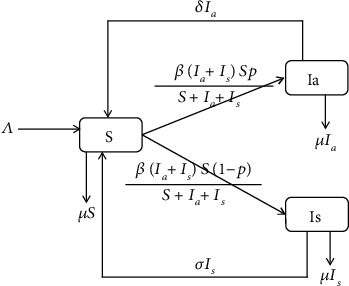
The transmission framework diagram of the SIS model.

Population was divided into: susceptible (*S*) and infected (*I*_*a*_, *I*_*s*_) before the establishment of a model. *I*_*a*_ presents nonclinic visits and *I*_*a*_ has infectivity, so they will generate new patients by contacting the susceptible [2, 3]. In addition, *I*_*a*_ can be recovered by autoimmunity and become susceptible (*S*) again. *I*_*s*_ means clinic visits, who must be cured after treatment and enter the susceptible (*S*) after recovery [4]. According to [2], there is almost no death due to infectious respiratory diseases, so death due to disease is not considered. The total population *N* satisfies *N* = *S* + *I*_*a*_ + *I*_*s*_. According to the transmission mechanism of respiratory diseases, the transmission framework diagram (Figure 2) and transmission model are as follows:

(1) $$\begin{array}{c} \left\{\begin{array}{c} \frac{dS}{dt}=\Lambda +\delta I_{a}+\sigma I_{s}-\frac{\beta \left(I_{a}+I_{s}\right)S}{S+I_{a}+I_{s}}-\mu S,\\ \frac{dI_{a}}{dt}=\frac{\beta \left(I_{a}+I_{s}\right)Sp}{S+I_{a}+I_{s}}-\left(\delta +\mu \right)I_{a},\\ \frac{dI_{s}}{dt}=\frac{\beta \left(I_{a}+I_{s}\right)S\left(1-p\right)}{S+I_{a}+I_{s}}-\left(\mu +\sigma \right)I_{s}. \end{array}\right. \end{array}$$

It is easy to know that *N*′ = *Λ* − *μN*. The invariant of system (1) is

(2) $$\begin{array}{c} D=\left\{\left(S,I_{a},I_{s}\right)\in R_{+}^{3}\;|\;0\leq S+I_{a}+I_{s}\leq \frac{\Lambda }{\mu },S\geq 0,I_{a}\geq 0,I_{s}\geq 0\right\}, \end{array}$$

where *Λ* means the constant population input rate, *β* is the contact infection rate of disease, and the incidence rate of disease is the standard incidence rate. *p* represents the proportion of nonclinic visits to the total number of patients. *μ* is the natural mortality rate. The rate of self-healing is *δ*, and *σ* is the cure rate.

Now, the next regeneration matrix [9] was used to calculate the reproduction number *R*_0_. We can get *F* and *V* from the system (1):

(3) $$\begin{array}{c} F=\left(\begin{array}{cc} \beta p, & \beta p\\ \beta \left(1-p\right), & \beta \left(1-p\right) \end{array}\right),\\ V=\left(\begin{array}{cc} \delta +\mu , & 0\\ 0, & \mu +\sigma \end{array}\right), \end{array}$$

where *F* is nonnegative and *V* is a nonsingular second-order matrix.

Then,

(4) $$\begin{array}{c} FV^{-1}=\left(\begin{array}{cc} \frac{\beta p}{\delta +\mu }, & \frac{\beta p}{\mu +\sigma }\\ \frac{\beta \left(1-p\right)}{\delta +\mu }, & \frac{\beta \left(1-p\right)}{\mu +\sigma } \end{array}\right). \end{array}$$

Therefore, the basic reproduction number is *R*_0_ = *ρ*(*FV*^−1^) = (*βp*/(*δ* + *μ*)) + ((*β*(1 − *p*))/(*μ* + *σ*)) = *R*_0*a*_ + *R*_0*s*_. *R*_0*a*_ means the average number of new infection caused by one *I*_*a*_ during the mean infectious period of *I*_*a*_. *R*_0*s*_ means the average number of new infection caused by one *I*_*s*_ during the mean infectious period of *I*_*s*_. *R*_0_ is the sum average number of total new infections produced by a single *I*_*a*_ and a single *I*_*s*_ during the mean infectious period.

### 2.2. Existence of Equilibriums

Let us consider the right hand of system (1) be 0 and *Λ* − *μN* = 0 to obtain the following equations:

(5) $$\begin{array}{c} \left\{\begin{array}{c} \Lambda +\delta I_{a}+\sigma I_{s}-\frac{\beta \left(I_{a}+I_{s}\right)S}{N}-\mu S=0,\\ \frac{\beta \left(I_{a}+I_{s}\right)Sp}{N}-\left(\delta +\mu \right)I_{a}=0,\\ \begin{array}{c} \frac{\beta \left(I_{a}+I_{s}\right)S\left(1-p\right)}{N}-\left(\mu +\sigma \right)I_{s}=0,\\ \Lambda -\mu N=0. \end{array} \end{array}\right. \end{array}$$

The following relations can be obtained from the second and third equations in the above equations:

(6) $$\begin{array}{c} S=\frac{\left(\delta +\mu \right)I_{a}}{\beta \left(I_{a}+I_{s}\right)p}N,\\ I_{a}=\frac{p\left(\mu +\sigma \right)}{\left(1-p\right)\left(\delta +\mu \right)}I_{s},\\ N=\frac{\Lambda }{\mu }. \end{array}$$

Then, the relations are brought into the first equation and become

(7) $$\begin{array}{c} I_{s}\left[\Lambda \beta p\left(\frac{p\left(\mu +\sigma \right)}{\left(1-p\right)\left(\delta +\mu \right)}+1\right)-\frac{\Lambda p\left(\mu +\sigma \right)}{\left(1-p\right)}\right]+I_{s}^{2}\left[\left(\frac{p\left(\mu +\sigma \right)}{\left(1-p\right)\left(\delta +\mu \right)}+1\right)\left(\delta \beta p\frac{p\left(\mu +\sigma \right)}{\left(1-p\right)\left(\delta +\mu \right)}+\sigma \beta p-\beta \frac{p\left(\mu +\sigma \right)}{1-p}\right)\right]=0. \end{array}$$

Obviously, *I*_*s*_ = 0 is a root of (7). Hence, disease-free equilibrium is

(8) $$\begin{array}{c} E_{0}=\left(S_{0},I_{a0},I_{s0}\right)=\left(\frac{\Lambda }{\mu },0,0\right). \end{array}$$

When *I*_*s*_ ≠ 0, let *A* + *I*_*s*_*BC* = 0, where

(9) $$\begin{array}{c} A=\Lambda \beta p\left(\frac{p\left(\mu +\sigma \right)}{\left(1-p\right)\left(\delta +\mu \right)}+1\right)-\frac{\Lambda p\left(\mu +\sigma \right)}{1-p},\\ B=\frac{p\left(\mu +\sigma \right)}{\left(1-p\right)\left(\delta +\mu \right)}+1,\\ C=\delta \beta p\frac{p\left(\mu +\sigma \right)}{\left(1-p\right)\left(\delta +\mu \right)}+\sigma \beta p-\beta \frac{p\left(\mu +\sigma \right)}{1-p}=\frac{\beta p}{\left(1-p\right)\left(\delta +\mu \right)}\left[\delta p\left(\mu +\sigma \right)+\sigma \left(1-p\right)\left(\delta +\mu \right)-\left(\mu +\sigma \right)\left(\delta +\mu \right)\right]. \end{array}$$

Since *B* > 0, let us consider the sign of *C*, we have

(10) $$\begin{array}{c} \delta p\left(\mu +\sigma \right)+\sigma \left(1-p\right)\left(\delta +\mu \right)-\left(\delta +\mu \right)\left(\mu +\sigma \right)=\sigma \left(1-p\right)\left(\delta +\mu \right)-\delta \left(1-p\right)\left(\mu +\sigma \right)-\mu \left(\mu +\sigma \right)=\left(1-p\right)\left[\sigma \left(\delta +\mu \right)-\delta \left(\mu +\sigma \right)\right]-\mu \left(\mu +\sigma \right)=\left(1-p\right)\left(\sigma \mu -\delta \mu \right)-\mu \left(\mu +\sigma \right)<\sigma \mu -\delta \mu -\mu \left(\mu +\sigma \right)=-\delta \mu -\mu^{2}<0. \end{array}$$

Thus, *C* < 0. Therefore, *BC* < 0. Since the endemic equilibrium exists if and only if *I*_*s*_ > 0, we consider *A* > 0, then

(11) $$\begin{array}{c} \frac{A}{p}=\Lambda \beta \left(\frac{p\left(\mu +\sigma \right)}{\left(1-p\right)\left(\delta +\mu \right)}+1\right)-\frac{\Lambda \left(\mu +\sigma \right)}{1-p}>0. \end{array}$$

We obtain

(12) $$\begin{array}{c} \frac{\beta p\left(\mu +\sigma \right)}{\left(1-p\right)\left(\delta +\mu \right)}+1>\frac{\Lambda \left(\mu +\sigma \right)}{1-p}. \end{array}$$

Therefore, endemic equilibrium *E*^∗^ = (*S*^∗^, *I*_*a*_^∗^, *I*_*s*_^∗^) exists if (*βp*/(*δ* + *μ*)) + ((*β*(1 − *p*))/(*μ* + *σ*)) > 1, i.e., *R*_0_ > 1. Where

(13) $$\begin{array}{c} S^{*}=\frac{\Lambda }{\mu }\frac{1}{R_{0}},\\ I_{a}^{*}=\frac{R_{0a}}{R_{0s}}I_{s}^{*},\\ I_{s}^{*}=\frac{\Lambda \left(1-R_{0}\right)}{\left(R_{0}/R_{0s}\right)\left(\delta R_{0a}-\sigma R_{0s}-\beta \right)}. \end{array}$$

**Remark 1 .**

From the above formula, note that *I*_*a*_^∗^ and *I*_*s*_^∗^ affect each other. Obviously, *I*_*a*_^∗^ > *I*_*s*_^∗^ for *R*_0*a*_ > *R*_0*s*_, i.e., *p* > ((*δ* + *μ*)/((*α* + *μ* + *σ*) + (*δ* + *μ*))). It shows that *p* will affect the final total number of patients.

## 3. Stability Analysis of Equilibriums

### 3.1. Local Stability Analysis of Equilibriums

**Theorem 1 .**

The disease-free equilibrium *E*_0_ of the system (1) is locally asymptotically stable if *R*_0_ < 1; *E*_0_ is unstable if *R*_0_ > 1.

**Proof**

The Jacobian matrix of the system (1) at the disease-free equilibrium *E*_0_ is

(14) $$\begin{array}{c} J_{E_{0}}=\left(\begin{array}{ccc} -\mu , & \delta -\beta , & \sigma -\beta \\ 0, & \beta p-\left(\delta +\mu \right), & \beta p\\ 0, & \beta \left(1-p\right), & \beta \left(1-p\right)-\left(\mu +\sigma \right) \end{array}\right). \end{array}$$

The secular equation corresponding to *J*_*E*_0__ is

(15) $$\begin{array}{c} \left|\lambda E-J_{E_{0}}\right|=\left|\begin{array}{ccc} \lambda +\mu , & -\delta +\beta , & -\sigma +\beta \\ 0, & \lambda -\beta p+\left(\delta +\mu \right), & -\beta p\\ 0, & -\beta \left(1-p\right), & \lambda -\beta \left(1-p\right)+\left(\mu +\sigma \right) \end{array}\right|=0, \end{array}$$

i.e.,

(16) $$\begin{array}{c} \left(\lambda +\mu \right)\left|\begin{array}{cc} \lambda -\beta p+\left(\delta +\mu \right), & -\beta p\\ -\beta \left(1-p\right), & \lambda -\beta \left(1-p\right)+\left(\mu +\sigma \right) \end{array}\right|=0. \end{array}$$

Let us consider

(17) $$\begin{array}{c} \left|\begin{array}{cc} \lambda -\beta p+\left(\delta +\mu \right), & -\beta p\\ -\beta \left(1-p\right), & \lambda -\beta \left(1-p\right)+\left(\mu +\sigma \right) \end{array}\right|=0, \end{array}$$

then

(18) $$\begin{array}{c} \lambda^{2}+\left[\left(\mu +\sigma \right)-\beta \left(1-p\right)+\left(\delta +\mu \right)-\beta p\right]\lambda +\left(\delta +\mu \right)\left(\mu +\sigma \right)-\beta \left(1-p\right)\left(\delta +\mu \right)-\beta p\left(\mu +\sigma \right)=0. \end{array}$$

Available from Vieta theorem, the roots of above equation satisfy

(19) $$\begin{array}{c} \lambda_{1}+\lambda_{2}=\beta \left(1-p\right)-\left(\mu +\sigma \right)+\beta p-\left(\delta +\mu \right),\\ \lambda_{1}\lambda_{2}=\left(\delta +\mu \right)\left(\mu +\sigma \right)-\beta \left(1-p\right)\left(\delta +\mu \right)-\beta p\left(\mu +\sigma \right). \end{array}$$

Note that *R*_0_ < 1 contains *R*_0*a*_ < 1 and *R*_0*s*_ < 1, then *λ*_1_ + *λ*_2_ < 0 and *λ*_1_*λ*_2_ > 0 if *R*_0_ < 1. Equation (15) has another eigenvalue *λ*_3_ = *μ* < 0, hence *λ*_*i*_ < 0, *i* = 1, 2, 3. Therefore, *E*_0_ is locally asymptotically stable.

Since *λ*_1_*λ*_2_ < 0 when *R*_0_ > 1, the secular equation must have one positive eigenvalue, then the disease-free equilibrium is unstable.

**Theorem 2 .**

The endemic equilibrium *E*^∗^ of system (1) is locally asymptotically stable.

**Proof**

The Jacobian matrix of the system (1) at the endemic equilibrium *E*^∗^ is

(20) $$\begin{array}{c} J_{E^{*}}=\left(\begin{array}{ccc} -\frac{\beta {\left(I_{a}^{*}+I_{s}^{*}\right)}^{2}}{{\left(\Lambda /\mu \right)}^{2}}-\mu , & \delta -\frac{\beta S^{*2}}{{\left(\Lambda /\mu \right)}^{2}}, & \sigma -\frac{\beta S^{*2}}{{\left(\Lambda /\mu \right)}^{2}}\\ \frac{\beta {\left(I_{a}^{*}+I_{s}^{*}\right)}^{2}}{{\left(\Lambda /\mu \right)}^{2}}p, & \frac{\beta S^{*2}}{{\left(\Lambda /\mu \right)}^{2}}p-\left(\delta +\mu \right), & \frac{\beta S^{*2}}{{\left(\Lambda /\mu \right)}^{2}}p\\ \frac{\beta {\left(I_{a}^{*}+I_{s}^{*}\right)}^{2}}{{\left(\Lambda /\mu \right)}^{2}}\left(1-p\right), & \frac{\beta S^{*2}}{{\left(\Lambda /\mu \right)}^{2}}\left(1-p\right), & \frac{\beta S^{*2}}{{\left(\Lambda /\mu \right)}^{2}}\left(1-p\right)-\left(\mu +\sigma \right) \end{array}\right). \end{array}$$

In order to prove that the eigenvalues of *J*_*E*^∗^_ are all negative, it is necessary to prove that *J*_*E*^∗^_ is a negative definite matrix. That is, for *J*_*E*^∗^_, the principal order of odd order is negative and the principal order of even order is positive [10].

Let

(21) $$\begin{array}{c} A_{1}=-\frac{\beta {\left(I_{a}^{*}+I_{s}^{*}\right)}^{2}}{{\left(\Lambda /\mu \right)}^{2}}-\mu <0,\\ A_{2}=\left|\begin{array}{cc} -\frac{\beta {\left(I_{a}^{*}+I_{s}^{*}\right)}^{2}}{{\left(\Lambda /\mu \right)}^{2}}-\mu , & \delta -\frac{\beta S^{*2}}{{\left(\Lambda /\mu \right)}^{2}}\\ \frac{\beta {\left(I_{a}^{*}+I_{s}^{*}\right)}^{2}}{{\left(\Lambda /\mu \right)}^{2}}p, & \frac{\beta S^{*2}}{{\left(\Lambda /\mu \right)}^{2}}p-\left(\delta +\mu \right) \end{array}\right|,\\ A_{3}=\left|J_{E^{*}}\right|. \end{array}$$

Let us consider

(22) $$\begin{array}{c} A_{2}=\left|\begin{array}{cc} -\mu , & \delta -\frac{\delta +\mu }{p}\\ \frac{\beta {\left(I_{a}^{*}+I_{s}^{*}\right)}^{2}}{{\left(\Lambda /\mu \right)}^{2}}p, & \frac{\beta S^{*2}}{{\left(\Lambda /\mu \right)}^{2}}p-\left(\delta +\mu \right) \end{array}\right|=-\mu \frac{\beta S^{*2}}{{\left(\Lambda /\mu \right)}^{2}}p+\mu \left(\delta +\mu \right)-\left(\delta -\frac{\delta +\mu }{p}\right)\frac{\beta {\left(I_{a}^{*}+I_{s}^{*}\right)}^{2}}{{\left(\Lambda /\mu \right)}^{2}}p. \end{array}$$

Since

(23) $$\begin{array}{c} \delta -\frac{\delta +\mu }{p}=-\frac{\left(1-p\right)\delta +\mu }{p}<0, \end{array}$$

we have −(*δ* − ((*δ* + *μ*)/*p*))((*βp*(*I*_*a*_^∗^ + *I*_*s*_^∗^)^2^)/(*Λ*/*μ*)^2^) > 0. Note that (1/*R*_0*a*_) > (1/*R*_0_^2^) if *R*_0_ > 1 and *S*^∗^ = (*Λ*/*μ*)(1/*R*_0_), hence

(24) $$\begin{array}{c} -\mu \frac{\beta S^{*2}}{{\left(\Lambda /\mu \right)}^{2}}p+\mu \left(\delta +\mu \right)=\mu \left[\left(\delta +\mu \right)-\frac{\beta S^{*2}}{{\left(\Lambda /\mu \right)}^{2}}p\right]=\mu \left[\left(\delta +\mu \right)-\frac{\beta p}{R_{0}^{2}}\right]=\mu \left(\frac{\beta p}{R_{0a}}-\frac{\beta p}{R_{0}^{2}}\right)=\mu \beta p\left(\frac{1}{R_{0a}}-\frac{1}{R_{0}^{2}}\right)>0. \end{array}$$

Therefore, *A*_2_ > 0. Through determinant transformation, we have

(25) $$\begin{array}{c} A_{3}=\left|\begin{array}{ccc} -\mu , & \delta -\frac{\delta +\mu }{p}, & \sigma \\ \frac{\beta {\left(I_{a}^{*}+I_{s}^{*}\right)}^{2}}{{\left(\Lambda /\mu \right)}^{2}}p, & \frac{\beta S^{*2}}{{\left(\Lambda /\mu \right)}^{2}}p-\left(\delta +\mu \right), & \frac{\beta S^{*2}}{{\left(\Lambda /\mu \right)}^{2}}p\\ 0, & \frac{1-p}{p}\left(\delta +\mu \right), & -\left(\mu +\sigma \right) \end{array}\right|=\left|\begin{array}{ccc} -\mu , & \delta -\frac{\delta +\mu }{p}, & \sigma \\ \frac{\beta {\left(I_{a}^{*}+I_{s}^{*}\right)}^{2}p}{{\left(\Lambda /\mu \right)}^{2}}, & \frac{\beta p}{R_{0}^{2}}-\left(\delta +\mu \right), & \frac{\beta p}{R_{0}^{2}}\\ 0, & \frac{1-p}{p}\left(\delta +\mu \right), & -\left(\mu +\sigma \right) \end{array}\right|=\mu \left(\mu +\sigma \right)\left[\frac{\beta p}{R_{0}^{2}}-\left(\delta +\mu \right)\right]-\sigma \left(\delta +\mu \right)\frac{\beta {\left(I_{a}^{*}+I_{s}^{*}\right)}^{2}\left(1-p\right)}{{\left(\Lambda /\mu \right)}^{2}}+\left(\mu +\sigma \right)\left(\delta -\frac{\delta +\mu }{p}\right)\frac{\beta {\left(I_{a}^{*}+I_{s}^{*}\right)}^{2}p}{{\left(\Lambda /\mu \right)}^{2}}+\mu \frac{\beta \left(1-p\right)}{R_{0}^{2}}\left(\delta +\mu \right). \end{array}$$

It is easy to know

(26) $$\begin{array}{c} -\sigma \left(\delta +\mu \right)\frac{\beta \left(1-p\right){\left(I_{a}^{*}+I_{s}^{*}\right)}^{2}}{{\left(\Lambda /\mu \right)}^{2}}+\left(\mu +\sigma \right)\left(\delta -\frac{\delta +\mu }{p}\right)\frac{\beta p{\left(I_{a}^{*}+I_{s}^{*}\right)}^{2}}{{\left(\Lambda /\mu \right)}^{2}}<0. \end{array}$$

Note that *R*_0*a*_ = *βp*/(*δ* + *μ*), *R*_0*s*_ = (*β*(1 − *p*))/(*μ* + *σ*), and *R*_0_ = *R*_0*a*_ + *R*_0*s*_, hence

(27) $$\begin{array}{c} \mu \left(\mu +\sigma \right)\left[\frac{\beta p}{R_{0}^{2}}-\left(\delta +\mu \right)\right]+\mu \frac{\beta \left(1-p\right)}{R_{0}^{2}}\left(\delta +\mu \right)=\mu \beta^{2}p\left(1-p\right)\frac{1}{R_{0}R_{0a}R_{0s}}\left(1-R_{0}\right)<0. \end{array}$$

Therefore, *A*_3_ < 0.

In conclusion, *J*_*E*^∗^_ is negative definite matrix. The proof is completed.

### 3.2. Global Analysis of Equilibriums

Before proving, the following lemma is introduced:

**Lemma 1 (see [11]).**

Suppose that *f* : [0, +∞)⟶*R* is bounded, quadratic differentiable and its second derivative are bounded. If *s*_*n*_⟶∞ and *f*′(*s*_*n*_)⟶0 when *n*⟶∞, then *f*(*s*_*n*_)⟶*f*^∞^, *n*⟶∞.

**Theorem 3 .**

The disease-free equilibrium *E*_0_ of system (1) is globally asymptotically stable if *R*_0_ < 1.

**Proof**

The disease-free equilibrium of system (1) is locally stable. The following proves that the disease-free equilibrium is globally attractive. Integrate and simplify the second and third equations of the system (1):

(28) $$\begin{array}{c} \left\{\begin{array}{cc} I_{a}\left(t\right)\leq e^{-\left(\delta +\mu \right)t}I_{a}\left(0\right)+\int_{0}^{t}e^{-\left(\delta +\mu \right)\tau }\beta p\left[I_{a}\left(t-\tau \right)+I_{s}\left(t-\tau \right)\right]d\tau , & \\ I_{s}\left(t\right)\leq e^{-\left(\mu +\sigma \right)t}I_{s}\left(0\right)+\int_{0}^{t}e^{-\left(\mu +\sigma \right)\tau }\beta \left(1-p\right)\left[I_{a}\left(t-\tau \right)+I_{s}\left(t-\tau \right)\right]d\tau . & \end{array}\right. \end{array}$$

We take that upper limit on both sides of the inequality, respectively,

(29) $$\begin{array}{c} I_{a}^{\infty }\leq \int_{0}^{t}e^{-\left(\delta +\mu \right)\tau }\beta p\left[\underset{t\to \infty }{\lim}\sup I_{a}\left(t-\tau \right)+\underset{t\to \infty }{\lim}\sup I_{s}\left(t-\tau \right)\right]d\tau =\frac{1}{\delta +\mu }\beta p\left(I_{a}^{\infty }+I_{s}^{\infty }\right),\\ I_{s}^{\infty }\leq \int_{o}^{t}e^{-\left(\mu +\sigma \right)\tau }\beta \left(1-p\right)\left[\underset{t\to \infty }{\lim}\sup I_{a}\left(t-\tau \right)+\underset{t\to \infty }{\lim}\sup I_{s}\left(t-\tau \right)\right]d\tau =\frac{1}{\mu +\sigma }\beta \left(1-p\right)\left(I_{a}^{\infty }+I_{s}^{\infty }\right). \end{array}$$

Adding the above two formulas, we obtain *I*_*a*_^∞^ + *I*_*s*_^∞^ ≤ *R*_0_(*I*_*a*_^∞^ + *I*_*s*_^∞^). If *R*_0_ < 1,

(30) $$\begin{array}{c} \underset{t\to \infty }{\lim}\left(I_{a}+I_{s}\right)=0\;\left(I_{a}\geq 0,I_{s}\geq 0\right), \end{array}$$

hence lim_*t*→∞_*I*_*a*_ = 0, lim_*t*→∞_*I*_*s*_ = 0. We select that *u*_*n*_⟶∞, *v*_*n*_⟶∞ so that *S*(*u*_*n*_)⟶*S*_∞_, *S*(*v*_*n*_)⟶*S*^∞^, and *S*′(*u*_*n*_)⟶0, *S*′(*v*_*n*_)⟶0. Then, it is obtained by the first formula of (30) and system (1)

(31) $$\begin{array}{c} \left\{\begin{array}{c} 0=\Lambda -\mu \lim\underset{t\to \infty }{\sup}S,\\ 0=\Lambda -\mu \lim\underset{t\to \infty }{\inf}S. \end{array}\right. \end{array}$$

We acquire

(32) $$\begin{array}{c} \underset{t\to \infty }{\lim}S\left(t\right)=\frac{\Lambda }{\mu }. \end{array}$$

Therefore, the disease-free equilibrium is globally attractive if *R*_0_ < 1. Thus, *E*_0_ is globally asymptotically stable.

In order to prove the global stability of the endemic equilibrium of the system (1), the following generalized Bendixson-Dulac theorem is introduced to exclude periodic solutions.

**Lemma 2 .**

Generalized Bendixson-Dulac Lemma [12].

Let *f* : *R*^3^⟶*R*^3^ be a *Lipschitz* continuous vector field and Γ(*t*) be a boundary curve of a directed smooth surface *S* ⊂ *R*^3^, which is closed and piecewise smooth. If $\overset{⟶}{g}:R^{3}⟶R^{3}$ is smooth in some field of *S* and for all *t*, $\vec{g}$ satisfies

(33) $$\begin{array}{c} \vec{g}\left(\Gamma \left(t\right)\right)\cdot \vec{f}\left(\Gamma \left(t\right)\right)\leq 0\left(\geq 0\right),\\ \left(\text{Curl}\vec{g}\right)\cdot \vec{n}\leq 0\left(\geq 0\right), \end{array}$$

in *S*, and some points on *S* satisfy

(34) $$\begin{array}{c} \left(\text{Curl}\vec{g}\right)\cdot \vec{n}>0\left(<0\right), \end{array}$$

where $\vec{n}$ is the unit normal vector on the surface *S*; then, Γ(*t*) cannot be composed of the trajectory of system *x*′(*t*) = *f*(*x*). The direction of Γ(*t*) and $\vec{n}$ forms a right-hand system.

**Lemma 3 (see [12]).**

Let *S* be a directed smooth surface. Γ(*t*) ⊂ *S* is an arbitrary smooth closed curve, and Γ(*t*) is the boundary of surface *S*′ ⊂ *S*. If *f* : Γ(*t*)⟶*R*^3^ is Lipschitz, *f* and *g* satisfy

(35) $$\begin{array}{c} \vec{g}\left(\Gamma \left(t\right)\right)\cdot \vec{f}\left(\Gamma \left(t\right)\right)=0,\\ \left(\text{Curl}\vec{g}\right)\cdot \vec{n}>0\left(<0\right), \end{array}$$

in *S*, where $\vec{n}$ is the unit normal vector on the surface *S*; then, Γ(*t*) cannot be the heteroclinic ring of *x*′ = *f*(*x*).

**Theorem 4 .**

There is no periodic solution for system (1).

**Proof**

Region *D* = {(*S*, *I*_*a*_, *I*_*s*_) | *S* ≥ 0, *I*_*a*_ ≥ 0, *I*_*s*_ ≥ 0, *S* + *I*_*a*_ + *I*_*s*_ ≤ (*Λ*/*μ*)} is the invariant set of system (1), and it is easy to obtain that the boundary of region *D* cannot be the periodic solution of system (1). Therefore, the following proof is discussed within region *D*.

Suppose that the system (1) has a periodic solution *Φ*(*t*) = {*S*(*t*), *I*_*a*_(*t*), *I*_*s*_(*t*)} in *D* and the plane region *Π* enclosed by the trajectory Ψ of *Φ*(*t*) is located inside *D*.

Let *f*_1_, *f*_2_, *f*_3_ be the expressions at the right end of equation (5), respectively. Consider $\vec{f}={\left(f_{1},f_{2},f_{3}\right)}^{T},\vec{g}\left(S,I_{a},I_{s}\right)=\left(1/SI_{a}I_{s}\right)\vec{q}\times \vec{f}$$\left(\vec{q}={\left(S,I_{a},I_{s}\right)}^{T}\right)$, obviously $\vec{g}\cdot \vec{f}=0$. Let $\vec{g}=\left(g_{1},g_{2},g_{3}\right)$ and $\text{Curl}\vec{g}=\left(\left(\partial g_{3}/\partial I_{a}\right)-\left(\partial g_{2}/\partial I_{s}\right),\left(\partial g_{1}/\partial I_{s}\right)-\left(\partial g_{3}/\partial S\right),\left(\partial g_{2}/\partial S\right)-\left(\partial g_{1}/\partial I_{a}\right)\right)$. After calculating, we have

(36) $$\begin{array}{c} \left(\text{Curl}\vec{g}\right)\cdot {\left(1,1,1\right)}^{T}=-\frac{\beta p}{S+I_{a}+I_{s}}\left(\frac{S}{I_{a}^{2}}+\frac{1}{I_{a}}+\frac{I_{s}}{I_{a}^{2}}\right)-\frac{\beta \left(1-p\right)}{S+I_{a}+I_{s}}\left(\frac{S}{I_{s}^{2}}+\frac{1}{I_{s}}+\frac{I_{a}}{I_{s}^{2}}\right)-\frac{1}{S^{2}}\left(\sigma +\delta \right)-\frac{1}{SI_{a}}\left(\sigma +\frac{\Lambda }{S}+\frac{\sigma I_{s}}{S}\right)-\frac{1}{SI_{s}}\left(\delta +\frac{1}{S}+\frac{\delta I_{a}}{S}\right)<0. \end{array}$$

Let the direction of *Π* be upward, and the direction of trajectory Ψ and the direction of *Π* form a right-hand rule. Since (1, 1, 1) is the normal vector of plane region *Π*, Theorem 4 is established by Lemma 2 and Lemma 3.

From the local asymptotic stability of *E*^∗^ and Theorem 4, the following theorem is obtained.

**Theorem 5 .**

The endemic equilibrium *E*^∗^ of system (1) is global asymptotically stable if and only if *R*_0_ > 1.

## 4. Numerical Simulation and Sensitivity Analysis

### 4.1. Numerical Simulation

The model parameters are *Λ*, *δ*, *σ*, *μ*, *p*, and *β*. The values of *Λ*, *δ*, *σ*, and *μ* can be acquired (see Table 1). Existed parameter values and daily cases of respiratory diseases in hospitals in Baohe District and Yaohai District of Hefei city from October 26, 2016, to October 25, 2017, were used for parameter estimation. The date selected here takes December 26, 2016, as the initial time, because the peak value and seasonal characteristics of the disease can be clearly observed from the data one year after this time. MCMC (Markov Chain Monte Carlo) was used to estimate unknown parameters *β*, *p* of system (1). The daily number of respiratory diseases was the value of *I*_*s*_. An accurate set of data of *I*_*a*_ was not available, since *I*_*a*_ had a strong resistance, and could be recovered itself through immunity so that it did not seek medical treatment. In the process of parameter estimation, through continuous simulation, the results showed that the initial value of *I*_*a*_ is 10-11 times of the initial value of *I*_*s*_. The fitting effect is well as *p* = 0.907.

Observing the actual data, it is easy to see that the first 100 data have obvious peaks; then, we took them out separately for parameter estimation. In the process of parameter estimation, the 100-day data can be further divided into two sections, 1-50 days and 51-100 days, respectively, with a lower peak in 1-50 days and a larger peak in 51-100 days. Therefore, parameter estimation was carried out on two sections, respectively, to obtain parameter *β* = 0.127729 for 1-50 days and parameter *β* = 0.1060537 for 51-100 days. The data of 101-297 days showed relatively flat, and there is no obvious peak trough characteristics, so the periodic function is used for fitting. The data of the last 289-365 days show a significant peak again; then, parameter estimation was used to obtain the parameter *β* = 0.1166635.

**Figure 3:**
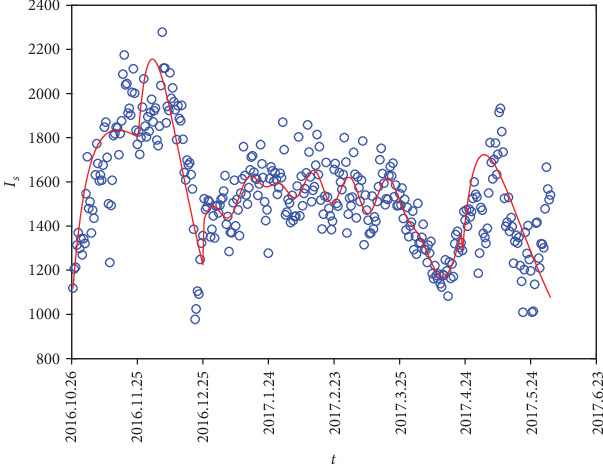
Fitting chart of *I*_*s*_ from October 26, 2016, to October 25, 2017.

After the above discussion, the corresponding parameter value was obtained, and the model was fitted to the actual data (as shown in Figure 3). As can be seen from Figure 3, the fitting effect is better. Accordingly, the model can be applied to respiratory disease data in the next year, which has a better practical significance. In addition, it can be seen from the figure that in winter (November to January), the daily number of respiratory diseases is at its peak, and the spread of the disease is relatively serious at this time. Then, during spring and summer (end of January to mid-August), the spread of respiratory diseases is relatively stable. Finally, a small outbreak of the disease occurs from early September to mid-September in autumn. Respiratory diseases have such seasonal characteristic, which may be related to the gathering of population in different seasons. There will be a large population gathering during the peak tourist season and holidays, which will easily lead to an increase in the number of respiratory diseases [14].

### 4.2. Sensitivity Analysis

**Figure 4:**
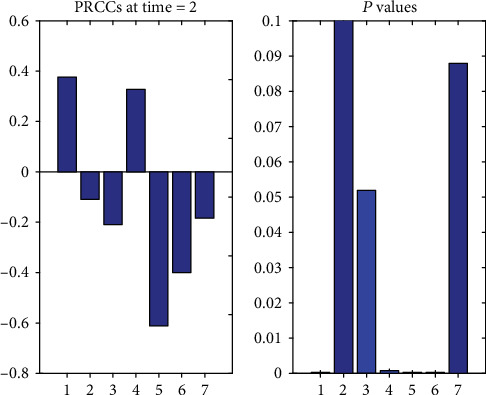
PRCC sensitivity analysis (1 is *Λ*; 2 is *δ*; 3 is *σ*; 4 is *β*; 5 is *μ*, 6 is *p*; 7 is dummy variable).

PRCC (Partial Rank Correlation Coefficient Sensitivity Analysis) was used to study the sensitivity of each parameter in system (1) to the number of clinic visits (*I*_*s*_). In Figure 4, the absolute value indicates the degree of influence of each coefficient on the output element, and the sign indicates the positive and negative correlation of each parameter with respect to the output element. A dummy variable reflects the influence of different attributes of different parameters on *I*_*s*_.

It can be seen from Figure 4 that the parameter that has the greatest effect on *I*_*s*_ is *μ*, followed by *p*, then *Λ*, *β*, *σ*, *δ*. Since the natural mortality rate *μ* and the constant population input rate *Λ* are uncontrolled factors, only the other four parameters are considered. Figure 4 shows us the following facts: *I*_*s*_ decreases with the increase of *p*, the decrease of *β*, and the increase of *σ* and *δ*.

**Figure 5:**
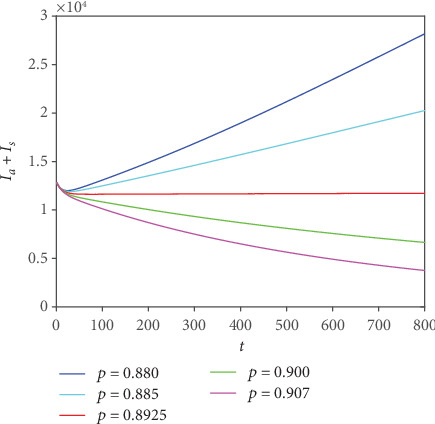
Trend chart of total patients corresponding to different *p* values.

We focus on the analysis of the influence of *p* on the total number of patients (*I*_*a*_ + *I*_*s*_). For this reason, we took 3 groups of different values of *p*; Figure 5 shows the development trend of the total number of patients.

We can see from Figure 5 that the limit is *p* = 0.8925 (it should be noted that the bound of this stationary state depends on the parameters and initial values). When the value of *p* is smaller, the final total number of patients (*I*_*a*_ + *I*_*s*_) is larger.

In order to observe the degree of increase (decrease) of the total number of patients with the decrease (increase) of *p* more clearly, we took 4 groups of data in the same step size at *p* > 0.8925 and *p* < 0.8925, respectively.

**Figure 6:**
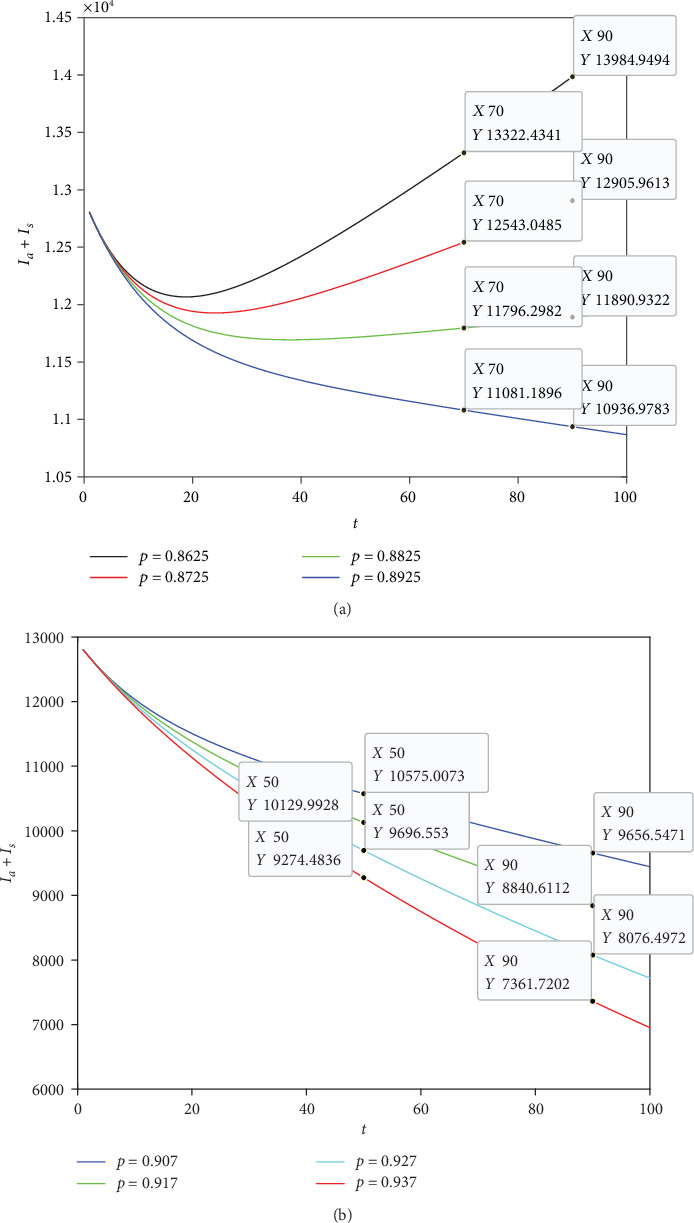
Influence of *p* value on relative growth (a) (decline (b)) of *I*_*a*_ + *I*_*s*_.

As can be seen from Figure 6, when *t* = 70, for every 1% decrease in *p*, *I*_*a*_ + *I*_*s*_ will increase by about 6 times (relative growth rate=(relative growth total number of patients)/(original total number of patients)×100%). When *t* = 90, for every 1% decrease in *p*, *I*_*a*_ + *I*_*s*_ increase by about 8 times. It is easy to see that when *t* increases, the degree of relative increase also increase. From Figure 6, when *t* = 50, for every 1% increase in *p*, the total number of patients decreases by about 4 times. When *t* = 90, for every 1% increase in *p*, the total number of patients decreases by about 8 times. When *t* increases, the relative decrease also increases. If *t* is fixed, *p* increases or decreases by 1%, and the total number of patients decreases or increases by a factor that is basically the same. However, with the increase of time *t*, the degree of relative increase or decrease will increase. Furthermore, if *p* is small enough, the total number of patients will drop significantly. The longer *t*, the faster the drop, which will be conducive to the control of disease transmission.

## 5. Conclusion and Discussion

Firstly, this article establishes the SIS model according to the transmission characteristics of respiratory diseases and the general situation of respiratory disease in Hefei. The equilibriums *E*_0_ and *E*^∗^ and the basic reproduction number *R*_0_ were obtained. It can be seen from the stability analysis results that when *R*_0_ < 1, *E*_0_ is globally asymptotically stable and *E*^∗^ is unstable, when *R*_0_ > 1, *E*^∗^ is globally asymptotically stable and *E*_0_ is unstable.

Secondly, the system was simulated with the daily number of respiratory diseases in Hefei. According to the general situation of the daily number of cases in Hefei from 2016 to 2017, the more representative time period data of 2016.10.26 to 2017.10.25 is selected, and the data is divided into 4 segments for numerical simulation according to the peak, trough, and seasonal characteristics of the data. Among them, MCMC parameter estimation is used for 1-50 days, 50-100 days, and 298-365 days to obtain the corresponding parameter values of each group.

In the results of parameter estimation and data simulation, the initial number of *I*_*a*_ was 10-11 times that of *I*_*s*_ was obtained. This conclusion is also valid in theory, because the solution of the system can be found, while the actual data represents the value of the solution of each point, and the value of the initial point can be obtained according to the symmetry of the solution about the initial value. In the final numerical simulation diagram of *I*_*s*_, it can be seen that the actual value is appropriate to the value simulated by the system, and the initial value of *I*_*a*_ obtained is reasonable and in line with reality. Accordingly, the fact that the number of nonclinic visits in the initial stage of epidemic transmission is huge cannot be ignored. Improving one's own resistance can help people be less susceptible to infections during an outbreak or recover as soon as possible after infection. In addition, according to the results of sensitivity analysis, it is also necessary to wear masks or isolate people with obvious symptoms. Reducing contact with the sick reduces the prevalence rate.

To sum up, the model of respiratory diseases in Hefei can be used to study the subsequent respiratory diseases in Hefei, as well as the outbreak time and degree of diseases in corresponding seasons. The number of nonclinic visits at the beginning of the outbreak can be estimated, which is undetectable in practice. Therefore, this article can help Anhui CDC to know more about the current situation of local respiratory disease transmission, pay attention to the early outbreak of respiratory disease in Hefei, and take preventive measures. People are called on to pay attention to improving their resistance so as to reduce the peak of the outbreak period and shorten the outbreak time.

## Figures and Tables

**Table 1:** Parameter value.

Parameter	Definition	Value	Reference
*Λ*	The constant population input rate	69.485 (day^−1^)	Data
*δ*	The rate of self-healing	0.142 (day^−1^)	[2, 13]
*σ*	The cure rate	0.07 (day^−1^)	[2, 14, 15]
*μ*	The natural mortality rate	3.7*e*-05 (day^−1^)	Data
*p*	The proportion of nonclinic visits to the total number of patients	0.89-0.91 (day^−1^)	Estimated
*β*	The contact infection rate of disease	0.105-0.128 (day^−1^)	Estimated

## Data Availability

The [Respiratory Disease] data used to support the findings of this study were supplied by Anhui CDC under license and so cannot be made freely available.

## References

[1] Stilianakis N. I., Drossinos Y. (2010). Dynamics of infectious disease transmission by inhalable respiratory droplets. *Journal of The Royal Society Interface*.

[2] Kharis M., Arifudin R. (2017). Mathematical model of seasonal influenza with treatment in constant population. *Journal of Physics: Conference Series*.

[3] Mizgerd J. P. (2012). Respiratory infection and the impact of pulmonary immunity on lung health and disease. *American Journal of Respiratory and Critical Care Medicine*.

[4] Meyer K. C. (2010). The role of immunity and inflammation in lung senescence and susceptibility to infection in the elderly. *Seminars in Respiratory and Critical Care Medicine*.

[5] Chen J., Yang F., Zhan S. (2003). Processing on the parameters and initial values of SARS simulation model for Beijing. *Acta Simulata Systematica Sinica*.

[6] Wang W., Ruan S. (2004). Simulating the SARS outbreak in Beijing with limited data. *Journal of Theoretical Biology*.

[7] Nainggolan J., Supian S., Supriatna A. K., Anggriani N. (2013). Mathematical model of tuberculosis transmission with reccurent infection and vaccination. *Journal of Physics: Conference Series*.

[8] Hsu S. B., Hsieh Y. H. (2008). On the role of asymptomatic infection in transmission dynamics of infectious diseases. *Bulletin of Mathematical Biology*.

[9] van den Driessche P., Watmough J. (2002). Reproduction numbers and sub-threshold endemic equilibria for compartmental models of disease transmission. *Mathematical Biosciences*.

[10] Ren C., Zhang P. (2014). The proof and nature of negative definite matrix. *Journal of Yili Normal University (Natural Science Edition)*.

[11] Thieme H. R. (1993). Persistence under relaxed point-dissipativity (with Application to an Endemic Model). *SIAM Journal on Mathematical Analysis*.

[12] Busenberg S., van den Driessche P. (1990). Analysis of a disease transmission model in a population with varying size. *Journal of Mathematical Biology*.

[13] Tangkanakul W., Tharmaphornpilas P., Thawatsupha P., Laolukpong P., Lertmongkol J. (2000). An outbreak of influenza a virus in a hilltribe village of Mae Hong Son Province Thailand, 1997. *Journal of the Medical Association of Thailand*.

[14] Xu F., McCluskey C. C. (2019). An investigation of the combined effect of an annual mass gathering event and seasonal infectiousness on disease outbreak. *Mathematical Biosciences*.

[15] Feng Z., Velasco-Hernández J. X. (1997). Competitive exclusion in a vector-host model for the dengue fever. *Journal of Mathematical Biology*.

